# Communicating serum chemical concentrations to study participants: follow up survey

**DOI:** 10.1186/1476-069X-9-20

**Published:** 2010-05-04

**Authors:** Alexandra J Buck, John E Vena, Bridget M McGuinness, Maureen A Cooney, Germaine M Louis

**Affiliations:** 1 University at Buffalo Law School, University at Buffalo, State of New York, John Lord O'Brian Hall, Buffalo, New York 14260, USA; 2 Department of Epidemiology & Biostatistics, College of Public Health, University of Georgia, 132 A Paul D. Coverdell Center, Athens, Georgia 30602, USA; 3 Department of Social & Preventive Medicine, School of Public Health & Health Professions, University at Buffalo, 270 Farber Hall, Buffalo, New York 14214, USA; 4 Division of Epidemiology, Statistics and Prevention Research, Eunice Kennedy Shriver National Institute of Child Health & Human Development, 6100 Executive Blvd., Rockville, Maryland 20852, USA

## Abstract

**Background:**

A considerable literature now supports the importance of effective communication with study participants, including how best to develop communication plans focusing on the uncertainty of health risks associated with particular environmental exposures. Strategies for communicating individual concentrations of environmental chemicals in human biological samples in the absence of clearly established safe or hazardous levels have been discussed from a conceptual basis and to a lesser extent from an empirical basis. We designed and evaluated an empirically based communication strategy for women of reproductive age who previously participated in a prospective study focusing on persistent environmental chemicals and reproductive outcomes.

**Methods:**

A cohort of women followed from preconception through pregnancy or up to 12 menstrual cycles without pregnancy was given their individual serum concentrations for lead, dichloro-2,2-bis*p*-chlorophenyl ethylene, and select polychlorinated biphenyl congeners. Two versions of standardized letters were prepared depending upon women's exposure status, which was characterized as low or high. Letters included an introduction, individual concentrations, population reference values and guidance for minimizing future exposures. Participants were actively monitored for any questions or concerns following receipt of letters.

**Results:**

Ninety-eight women were sent letters informing them of their individual concentrations to select study chemicals. None of the 89 (91%) participating women irrespective of exposure status contacted the research team with questions or concerns about communicated exposures despite an invitation to do so.

**Conclusions:**

Our findings suggest that study participants can be informed about their individual serum concentrations without generating unnecessary concern.

## Background

Successful research requires mutual respect and trust between investigators and study participants, and adherence to the highest standards of research ethics on the part of researchers. Implicit in this premise is an obligation to communicate exposures and risks that become known as a part of the research initiative. Such expectations are grounded within the tenets of research ethics, viz., ensuring autonomy or an individual's right to know along with risks and benefits being equitably distributed to study participants [1,2]. Professional societies such as the International Society for Environmental Epidemiology also encourage the broad communication of results including to affected communities [3].

In the context of environmental health, much of the risk communication literature has historically focused on the release of aggregate exposure data to communities rather than the release of individual data [4]. Even within communities, individual risk may vary and study participants may largely be interested in their risk, *per se*. Scientists may be reluctant to discuss individual risks, even if such information could be accurately obtained, given the uncertainty of the information being communicated with regard to human health risks [5]. To address these and other issues, participatory research forums have evolved and gained the interest and respect of researchers, communities and companies as evidence by the formation and work of early community advisory panels [6-8]. In an attempt to minimize the "communication gap" between experts and the public [9], researchers and industrial managers were encouraged to view themselves as partners with the public so that communication becomes an interactive dialogue and interactive process of information exchange and not strictly a one-way conversation [8,10]. In fact, some industries require a communication plan for all protocols [11].

Of late, an evolving avenue of research has developed conceptual frameworks for communicating results to study participants. In response to the 2006 National Academy of Sciences' *Human Biomonitoring for Environmental Chemicals *report calling for more attention to communicating data [12], Morello-Frosch and colleagues [13] discussed three frameworks for communicating biomonitoring results to study participants. Briefly, these include: 1) a clinical ethics framework in which exposures with a known clinical action or health effect relation are reported to participants; 2) a community based participatory research framework that respects study participants right to know from study design and inception; and 3) a citizen-science data judo that encourages communicating individual or aggregate results from an advocacy or precautionary perspective. Irrespective of framework, it is important to note the longstanding social sciences literature that has long recognized the importance of appropriately disseminating innovations including ideas to target populations [14], contrasting with the lag in addressing this issue on the part of environmental scientists. Another challenge in designing communication strategies is in deciding what to communicate and how. Suggestions include understandable language and guidance for any required action [15]. Other investigators identified a series of questions that reflect their experience in reporting back individual levels including what and how much was found, high/low or safe values, what one should do, where exposures come from and any recommendations [16]. Recognizably, the ideal communication strategy is developed for the study population at the design phase of research and remains an integral aspect throughout every aspect of the study. In fact, in 1995 the National Institute of Environmental Health Sciences (NIEHS) launched the Environmental Justice: Partnerships for Communication Program to promote community based participatory research and environmental justice [17,18] with subsequent support from the Environmental Protection Agency and National Institute for Occupational Safety and Health. This aim of the NIEHS program was to foster greater community involvement in all aspects of research by bringing three partners together: 1) a community organization; 2) an environmental health researcher and 3) a health care professional for developing and fostering communication to increase community participation in the research process. From a pragmatic perspective, however, funding and other logistical considerations may not readily support Community Based Participatory Research (CBR) platforms for all environmental research even when desired, requiring investigators to seek other venues for communication. Another challenge is the receipt of exposure data late in the research process or even after funding timelines have ended resulting in challenges for how best to communicate exposure information once the research is completed. Essentially, the research team may have two options-to ignore the expectation to give results back or to seek strategies for communicating results upon completion of the study recognizing that the approach is less than ideal.

Empirical evaluation of the content of communication strategies is limited [16,19] providing little published evidence in support of investigators' efforts to convince Institutional Review Boards (IRBs) or other entities of the need to communicate individual values in the context of uncertain health effects and risks. Altman and colleagues conducted 30 qualitative interviews with 25 women who received exposure data as a part of their participation in the Household Exposure Study [19]. Among other key findings, the authors noted that participants reported wanting their results including technical explanations about what is or is not known. Moreover, the participants did not react with alarm. Morello-Frosch and colleagues [13] interviewed 26 scientists, IRB officials and study participants and reported that most individuals stated it was desirable to set expectations before commencing data collection and communication protocols. Also, reporting individual levels can serve as an impetus for individual action to minimize exposures. To this end, researchers have few available data to help decide if, when and how individual concentrations should be reported to study participants, and how best to communicate the uncertainty of possible health risks. In our collective experience, IRBs have varying positions regarding whether or not to communicate individual concentrations when health effects are unclear consistent with other reports [13]. This may reflect the uncertainty associated with what constitutes "safe" concentrations or our uncertain understanding of associated health effects. Moreover for persistent environmental toxicants, there may be little study participants can do to lower their concentrations in an effort to maximize their health. However, knowledge of their individual levels may inform partipants'decisions about future health behaviors.

Thus, the many critical data gaps underlying the relation between environmental chemicals and human health coupled with limited ways to minimize exposure are important considerations in devising a strategy for communicating individual concentrations to study participants even if delivered long after completion of the study. To this end, we designed and evaluated a strategy for communicating lead, dichloro-2,2-bis*p*-chlorophenyl ethylene (DDE) and select polychlorinated biphenyl (PCB) congener concentrations to women who participated in a prospective pregnancy study aimed at identifying the potential reproductive and/or developmental toxicity of these persistent environmental contaminants. We sought to communicate individual concentrations of persistent environmental contaminants to study participants in the context of little to no resources available for doing so in keeping with our original promise to deliver such information.

## Methods

In 1996-1997, 1,031 women who had previously participated in a cross-sectional study focusing on health status in relation to consumption patterns of contaminated sport fish from Lakes Erie and Ontario and other tributaries in Upstate New York [20] were re-contacted and invited to participate in a prospective pregnancy cohort study. The purpose of this latter study as communicated to women was to assess the potential reproductive toxicity of PCBs and metals. Seventy-six percent of women (n = 787) were ineligible for the following reasons: 1) above 44 years of age, 2) had a physician diagnosis of infertility or 3) were not planning a pregnancy in the next few months. Among the 244 eligible women, 131 refused participation while 113 (46%) agreed to participate. The final cohort comprised 99 women after excluding 14 women who were already pregnant at the time of study enrollment. Women were recruited upon discontinuing contraception for purposes of attempting to become pregnant and followed until a positive home pregnant test result or up to 12 menstrual cycles with intercourse during the fertile window. Participation in the study was intensive and required a baseline interview, completion of a daily diary on lifestyle factors purported to affect time-to-pregnancy (i.e., use of cigarettes, alcoholic and caffeinated beverages, and fish consumption) and blood donation at sensitive windows (i.e., preconception, following a positive pregnancy test, following a pregnancy loss or delivery, or after 12 months without conception). A more complete description of the protocol is provided elsewhere [21,22]. Upon recruitment, women were told they would receive their individual concentrations to select compounds, but were cautioned that it would most likely take several years. It was necessary to note this caution as the research team worked with the IRB to include this component, and given concerns at the state health department level about what could or could not be communicated to study participants. During the time frame planning and conducting this study, there was a generalized recognition of the need to communicate results but little consensus regarding strategy. For all aspects of the study including the prospective pregnancy cohort component, full human subjects approval was obtained and all women were consented prior to participation in the study.

In 2006, addresses for the original study cohort (n = 99) were updated and women were sent postcards informing them that our research team would be sending their chemical concentrations to them along with one last request for completion of a self administered questionnaire (SAQ) aimed at assessing how well women recall what they were doing while attempting to become pregnant. A subsequent mailing was prepared and included an introductory letter printed on the study's original letterhead and sent to 98 women; one woman was deceased.

A two-page letter was designed with the first page being personalized to the women and providing introductory remarks and the second page containing the data on serum concentrations of contaminants and guidance for exposure avoidance. Specifically, letters included an introduction with detailed contact information for the investigator (JEV) dedicated to discussing results, concentrations of serum DDE and PCB congeners and blood lead for each woman along with two comparisons concentrations (including one for a representative U.S. female population aged ≥ 12 years [23] and a fish eating population), and recommendations and guidance for minimizing exposure largely in relation to fish consumption as developed by the state health department. It is important to note that the prospective pregnancy cohort was obtained from the overall New York State Angler Cohort as defined by purchase of a fish and/or hunting license.

With regard to exposures, each letter contained a standardized 5-column table that first listed the name of the environmental contaminants, the woman's individual concentration upon enrollment into the cohort, mean concentrations for two referent populations, and a column noting that lead was the only contaminant for which a known concentration was indicative of a health alert (see Table 1). *A priori*, six compounds and a sum of all measured PCB congeners were selected to be reported back based upon the following considerations: 1) exposures believed relevant for the angler sampling framework; 2) input from participating toxicologists and analytic chemists responsible for quantification; and 3) guidance from collaborating state health department investigators, particularly with regard to established health alerts. Specifically, women were given their individual serum concentrations of DDE and PCB congeners 118, 138_158, 153, 180, and a simple sum of all 76 measured PCB congeners (ng/g lipids) and blood lead (μg/dL). The PCB sum was provided as a measure of overall PCB exposure as measured by our laboratory. All PCB and DDE concentrations were expressed in ng/g lipids for comparison purposes. Women whose concentrations were below the laboratory limits of detection (LOD) were told that their serum concentrations were at or below those usually found in other groups of people, per the decision made by the scientific disciplines represented on the original research team. Our study cohort essentially had background exposures. The mean blood lead level for our cohort was 1.6 μg/dL compared to 4.6 μg/dL in NHANES [23]. Of the six women with concentrations above any of the referent values or those who received the standardized high letter, we attached a copy of the most recent state issued guidance to the letter and provided a website for additional information as released by the State Health Department. Five women had serum PCB 153 concentrations higher than the U.S. reference, but lower than that reported for fish eaters. One woman had a higher PCB 118 concentration than that reported for the U.S. reference but lower than for anglers. The woman's letter contained the contact information (mail and email addresses and telephone number) for one of the study's investigators (JEV) who agreed to serve as the contact person for the study. This individual was one of the original study investigators and his name has appeared on all communication with the full and pregnancy cohorts since inception in 1991.

**Table 1:** Illustration of the Data Format Used for Personalized Letters

Chemical	Woman's Concentration	U.S. Reference Concentration	Fish Eater Population Concentration	Health Based Concentration
Lead (μg/dL)				25 μg/dL

DDE (ng/g lipid)				

PCB118 (ng/g lipid)				

PCB138/158 (ng/g lipid)				

PCB153 (ng/g lipid)				

PCB180 (ng/g lipid)				

Total all PCB Congeners (ng/g lipid)				

DDE refers to dichloro-2,2-bis*p*-chlorophenyl ethylene

PCB refers to polychlorinated biphenyls

The dissemination plan included monitoring all queries received from women and associated outcomes. A second study aim was to ask women to complete a short self-administered questionnaire (SAQ) asking them to report their usual lifestyle behaviors while attempting to become pregnant that were originally included in the daily diaries that were used in the prospective pregnancy study. The intent of the questionnaire was to formally assess the validity of self reported exposures during the sensitive periconception window. Women were told that we were attempting to determine how well women remember what they were doing while attempting to become pregnant [24]. This SAQ provided women with another implicit forum for asking questions about any aspect of the original or follow up study including questions about their exposures.

## Results

Eighty-nine women (91%) returned completed questionnaires a decade after originally participating in an intensive prospective pregnancy cohort study with preconception enrollment. This signified to us that the vast majority of women actually received the letters communicating their individual concentrations. We did not follow up the 9% of women not returning questionnaires, given that our IRB approval only covered the dissemination of their laboratory data and one last querying of women about behaviors while attempting pregnancy in light of the amount of study participation throughout all aspects of the study. Also, chemical concentrations were not significantly associated with women's participation status in the communication study.

None of the women ever contacted the designated contact person at any time since the mailing of letters. Nor was any other member of the research team contacted in any way including the study coordinator (BMM) with whom most women had close working relationships while participating in the intensive prospective pregnancy cohort study and followup of children up to age 2 years. We assumed that such a relationship would have facilitated women calling with questions or concerns regarding their exposures, though this was only an assumption. None of the women used the enclosed questionnaire to note concerns or questions about their exposures. They did offer, however, *ad lib *comments about their fertility or children recognizing the purpose of the pregnancy cohort study.

## Discussion

The dissemination of individual concentrations of select persistent environmental chemicals to women participating in a prospective pregnancy study did not introduce any known harm, at least none known to the research team. Specifically, we received no calls or letters with questions or asking for further information or assistance in any manner despite our expectation and planning for such inquires. The absence of comments about individual exposures from women may suggest that they were satisfied with the type and amount of information and that they had no additional questions regarding possible health effects (including reproductive). This may reflect, in part, our longstanding partnership with the women over a decade or, possibly, reluctance on the part of women to contact the research team regardless of reason. Given that women readily contacted the research team as a part of the original prospective pregnancy cohort study, largely for questions pertaining to scheduling or compliance with the protocol, we suspect that reluctance was not the reason for a lack of follow up calls or email messages. It is also important to note that we were not without contact of women during the 10-year interval. In fact, 96% of women with live births enrolled their offspring in a prospective child follow up study through age two years [25]. As a part of this follow up study, children's blood lead and thyroid levels were communicated to parents as soon as values were available from the participating clinical laboratory (usually months). None of the infants had levels with reportable action, and none of the parents contacted the research office with questions or concerns.

The absence of comments or questions from the women following receipt of their concentrations should not be interpreted as being equivalent to a lack of harm in so doing, since we were not able to follow up the women to formally assess comprehension or outstanding questions or concerns. We recognize this study limitation and urge readers to cautiously interpret of our findings. A second limitation impacting the interpretation of results is that we have no information on whether woman sought additional information from sources provided in the letters or elsewhere. The educational status of women was high with 88% of women having attended/graduated from college, and high letters were sent to only six women. The extent to which this approach may be successful for women with lower educational attainments remains to be established.

It is likely that other investigators have communicated individual concentrations for various environmental chemicals to study participants but never published their experiences. In an effort to further develop an empirical database for such communication, we formalized our communication strategy and results to the extent possible. We do, however, recognize that our efforts are far from ideal in that we were unable to devise a community based participatory research paradigm at the time the study was designed and implemented, the lack of personal contact in communicating results despite efforts to personalize each letter and to ground the letters within the original study, and the absence of any formal evaluation of comprehension of information communicated. Still, our findings may prompt investigators to provide individual exposures in a concerted manner even when resources are limited or when data become available as funding ceases.

The approximately 10-year interval between the women's initial participation in the study and receipt of her individual concentrations is less than ideal. A number of factors were responsible including the time required for analyzing and quantifying the samples in an era when the laboratory technology was much slower than contemporary practice, the time required in obtaining support from the health department for communicating individual levels to study participants, a funding interruption, and the professional relocation of two study investigators. While we are unable to empirically assess how length of time may have affected our communication plan, suffice to say that we believe we had established a rapport with the cohort as evidence by the high response rate for the SAQ enclosed with the letter communicating their results. Lastly, the ability to provide study participants with results even after 10 years may prompt investigators who have yet to provide such information to do so.

As research efforts continue to support the ability of researchers to communicate results to study participants using a variety of approaches, it will be necessary to build an empirical database that reflects the strengths and weaknesses of the various approaches. Despite our efforts using conventional search techniques for accessing the literature, we were unable to identify randomized community intervention designs aimed at the empirical evaluation of two or more risk communication strategies. Perhaps, such data will become available in the near future as the importance of communicating exposures and results becomes mainstream.

## Conclusions

Our findings suggest that communicating individual blood or serum concentrations of selected environmental chemicals to study participants is feasible without introducing untoward events, worry or other stressors as measured by the absence of telephone calls or written messages indicative of concern or uncertainty. We support efforts to actively develop communication strategies at the design phase of research and to empower study populations in this process. As original research initiatives aimed at answering questions about environmental contaminants and human health increase, a growing number of individuals or communities are being asked to participate in research and, thereby, have a stake in the findings. Efficient, effective and time sensitive communication strategies should be a mainstay of environmental epidemiology along with their empirical evaluation to ensure the public is being served.

## Abbreviations

DDE: dichloro-2,2-bis*p*-chlorophenyl ethylene; IRB: Institutional Review Board; LOD: limits of detection; PCB: polychlorinated biphenyl; SAQ: self administrated questionnaires.

## Competing interests

The authors declare that they have no competing interests.

## Authors' contributions

AJB, MC and BMM were involved in framing the question for this paper and researching the literature; BMM was responsible for the distribution of letters and follow up with study participants; JEV and GL were investigators on the project and were responsible for all aspects of the study. All authors were involved in writing the paper and all take public responsibility for its content. All authors read and approved the final manuscript.
